# Unmasking quality: exploring meanings of health by doing art

**DOI:** 10.1186/s12875-015-0233-x

**Published:** 2015-02-27

**Authors:** Moira Kelly, Carol Rivas, Jens Foell, Janet Llewellyn-Dunn, Diana England, Anna Cocciadiferro, Sally Hull

**Affiliations:** Centre for Medical Education, Institute of Health Sciences Education, Queen Mary University of London, Room 2.10, Garrod Building, Turner Street, Whitechapel, London E1 2AD UK; University of Southampton, School of Health Sciences, Highfield, Building 67, Southampton, SO17 1BJ UK; Centre for Primary Care and Public Health, Blizard Institute, Queen Mary University London, Yvonne Carter Building, 58 Turner Street, Whitechapel, London E1 2AB UK; Stitches in Time, Old Limehouse Town Hall, 646 Commercial Road, London, E14 7HA UK

**Keywords:** Primary care, Art, Social prescribing, Community development

## Abstract

**Background:**

Quality in healthcare has many potential meanings and interpretations. The case has been made for conceptualisations of quality that place more emphasis on describing quality and less on measuring it through structured, vertically oriented metrics. Through discussion of an interdisciplinary community arts project we explore and challenge the dominant reductionist meanings of quality in healthcare.

**Discussion:**

The model for structured participatory arts workshops such as ours is ‘art as conversation’. In creating textile art works, women involved in the sewing workshops engaged at a personal level, developing confidence through sharing ideas, experiences and humour. Group discussions built on the self-assurance gained from doing craft work together and talking in a relaxed way with a common purpose, exploring the health themes which were the focus of the art. For example, working on a textile about vitamin D created a framework which stimulated the emergence of a common discourse about different cultural practices around ‘going out in the sun’. These conversations have value as ‘bridging work’, between the culture of medicine, with its current emphasis on lifestyle change to prevent illness, and patients’ life worlds. Such bridges allow for innovation and flexibility to reflect local public health needs and community concerns. They also enable us to view care from a horizontally oriented perspective, so that the interface in which social worlds and the biomedical model meet and interpenetrate is made visible.

**Summary:**

Through this interdisciplinary art project involving academics, health professionals and the local community we have become more sensitised to conceptualising one aspect of health care quality as ensuring a ‘space for the story’ in health care encounters. This space gives precedence to the patient narratives, but acknowledges the importance of enabling clinicians to have time to share stories about care.

**Electronic supplementary material:**

The online version of this article (doi:10.1186/s12875-015-0233-x) contains supplementary material, which is available to authorized users.

## Background

Penelope sews:

A god gave me the inspiration to set up a great web on my loom in my house, and begin weaving a large and delicate piece of work. “My suitors, now that noble Odysseus is dead, restrain your ardour, do not urge on this marriage till I have done this work, so the threads I have spun may not be wasted”. That is what I said, and they magnanimously consented. So by day I used to weave the great web, but every night had torches set and undid the work. For three years I took them in by this stratagem [1].

Despite contemporary ‘patient-centred’ healthcare being premised on the bio-psychosocial model, the ‘bio’ aspects are usually foregrounded when healthcare quality is considered, dominating the other elements [2]. This reductionist approach means that the role of the clinician as agent of change is ignored, with doctors driven to pigeonhole healthcare activities into discrete tasks that can easily be measured against explicit, external standards. The resulting match of performance to standards can be easily monitored, providing a form of ‘quality’ measure that tightly defines process and outcome, but may have the particular needs of the patient and the context of the clinician-patient relationship stripped away. Psychological and social elements recede, and the potentially important intersections between the three elements are not brought into view. When biomedical and physical processes become dominant medicine is at risk of becoming dehumanised; the biomedical and the physical process take priority over the quality of human interaction, including the social, psychological and cultural. This then affects the language we use about healthcare, which is often diminished to the objective, ignoring the narrative and interpretive function of medicine [3,4]. Restricting the range of language in turn subtly restricts the way we think about healthcare, influencing how care quality (including decision making) is viewed and targeted. The role of the clinician as agent of change is displaced, with the space for interaction with patients occupied by the language of safety, efficiency and the patient as consumer, representative of the unchallenged hegemony of the market in healthcare.

Standardised metrics for quality of life, the length of life or years in good health gained, form the currency for what matters for the purchasers of healthcare in the UK National Health Service (NHS). This care is vertically oriented, concerned with specific diseases or conditions [5], with health metrics summarised for conditions (or institutions or regions).^a^ Hence when we think about what is excellent, or of high quality, we are often constrained only to consider what can be measured. This paradigm does not encompass many of the problems that are presented in general practitioners’ surgeries [6,7]. Patients present symptoms and experience ‘illness’, which ‘stands for a patient’s interpretation of his or her disease, the feelings that accompany it, the life events it turns into’ ([8], p.9]. For care to be patient-centred, vertically oriented care needs to be balanced with horizontally oriented care which works to integrate the often complex needs of individuals with care systems that are responsive to the needs of local populations [5]. Primary care is the interface in which social worlds and the biomedical model meet and interpenetrate, an intersection which requires considerable negotiation at many different levels. Space is needed for stories to emerge and be refashioned, both those of patients in consultations and those of the clinicians engaged in complex negotiations around care and what might be seen as good care from different perspectives.

### The art project

We engaged in an arts project which involved the design and production of three textile art works for the university centre for primary care and public health in east London. The department art committee chose to seek out art works to reflect the community location of the department and its work on health. A joint funding application was made with a local community arts project, ‘Stitches in Time’, with the plan of co-creating textile hangings reflecting the community and research department perceptions of local health problems. As the purpose of the project was specifically to produce art works to be displayed, rather than being a research project involving an evaluation, ethical approval was not sought. This discussion paper has emerged from our reflections on the meaning of quality in healthcare during the process of producing the art.

Initial workshops involving departmental researchers and the artists from Stitches in Time identified three themes as being central to the current research work of the department, namely, vitamin D, diabetes prevention and physical activity. The Stitches in Time team used these ideas to explore designs in sewing workshops with local women. The workshops were drawn from existing community groups and parent groups linked to local schools, and largely included women from the local Bangladeshi community.^b^

The art designs were developed iteratively over the six weekly sessions of the sewing groups, led by the artist who brought materials and information about the health themes to the table. The way in which the process of producing the art was necessarily interwoven with discussions about health, which was the focus of the textiles, is described by the Stitches in Time team below. By popular request a sewing workshop was also held in the department, allowing the local women working on the textiles to teach departmental staff sewing techniques and sharing something of the health conversations which ensued (Additional file 1: Photograph 1).

*Producing a collaborative artwork with a group is a process that mirrors the practice and process we engage in for our own work as textile designers. For this project we had a clear purpose, to design and produce a textile wall-hanging around health themes. The approach taken wa one of experimentation, exploration and reflection at each stage.*

*We started with our visual research, our subject matter, related to the health theme we were working with. For the art work on ‘vitamin D’ this took the form of a ‘‘healthy still life scene’’ including fresh fish, sunflowers to symbolise sunlight, tins of canned oily fish, and cartons of eggs. Materials and art techniques were used to explore the subject matter in a way that resonated with the individuals in the groups. Using observational drawing and mono-printing we experimented with different marks and gestures, using paint and pencils to explore our responses to this ‘‘still life’’.*

*Our conversations explored and made another level of meaning around what the fish meant to us in terms of our diets, with the sunflowers prompting discussions around sunlight and its impact on our health. People’s personal stories surfaced and evolved through engagement in the workshops, experiences were linked and questions asked and reflected upon. At the same time as talking about how we could make easy cheaper meals using tinned oily fish, we looked at the designs and illustrations on sardine tin packaging and understood from our own drawings how these illustrative designs emerged and how we could translate this to our design process for the textile artwork.*

*We engaged in multi-layered explorations and group dialogue around the health themes which were the focus of the project. These were the over-arching feature that was consistent throughout the whole process. As the groups developed and refined their initial research to create the art works using textile techniques, visual ideas both expanded in terms of experimentation and boldness, and at the same time focussed in on detail of stitch and pattern. Our conversations around the health themes too followed a similar pattern of opening out, sharing and a moving towards a closer understanding of details that could have an important impact.*

*Stitches in Time team (JL-D, AC, DE)*

Our aim in this paper is to use the reflections and insights gained from working on the interdisciplinary community arts project to explore and challenge the dominant reductionist meanings of quality in health care.

## Discussion

### Art as conversation

Encounters at structured participatory arts workshops involve experiential learning, including awareness about the topics of the art produced, which in our case was health. This is ‘art as conversation’ (in contrast to ‘art as therapy’), and implies relationships of equality and a blurring between the role boundaries of those involved [9]. Conversation is a starting point, engaging people at a personal level and developing confidence through sharing ideas, experiences, and humour. This was evident in the conversations that arose about health and illness in our workshops. One example was the provision of visual stimuli to explore everyday access to Vitamin D, leading to discussions in the sewing groups of how this knowledge could be communicated to others through the textiles.

The main task of the sewing sessions was to design and make high quality bespoke art works. This established community arts approach involved a social contract between the textile designers and community members to commit guided time to working towards a product designed and produced together. In our project, through the effective use of the group process, the sessions also enabled a sense of achievement by solving design ideas through drawing, printing and the intricate repetitive tasks of lace making or embroidery. Group discussions built on the self-assurance gained from doing craft work together and talking in a relaxed way with a common purpose. Health issues were raised and explored informally as can be seen below in the description of a sewing group conversation about healthy eating.

*....there was a computer and projector screen in the room. I often went online quickly to show visuals to enhance our learning. In one session we were chatting about fried foods. Two women were diabetic and brought up how important it is to be aware of high levels of saturated and hydrogenated fats in processed foods. The conversation turned to chicken nuggets, leading to a search on youtube of how they are made in factories. This was followed by questions about how commercial kebabs (commonly available in the local area) were made. The room exploded with strong reactions of disgust to the youtube clip of how kebabs were made. A couple of women took notes with plans to show the clip to their families. (JL-D)*

Contributions to well-being arose via the emphasis on ‘understanding through doing’, by exchanging questions and answers about processed food, by demonstrating that a group experience can lead to individual action such as showing the family some of the facts discovered, swapping cooking tips and recipes, and exploring ways of exposing children to more sunlight. It was endorsed weekly by sharing achievements and discussion alongside activity. Further benefits arose when sewing group members celebrated their success in producing the art works, seeing them in the wider context of the academic health department where the textiles are located. Links were made between the community and local researchers building social capital through helping people see the value of their skills, and through conversations, many of which were necessarily transcultural. We held a lively social event to ‘unveil’ the art works attended by the whole of our interdisciplinary team, including many of the women from the sewing groups (Additional file 2: Photograph 2).

Critical factors to the success of arts for health projects include creating high quality work with a purpose, and good leadership and organisation to ensure this happens [9,10]. This is in contrast to what happens when art is primarily used as therapy. In this project we have created objects of value, and linked to a sewing heritage which is at risk of becoming marginalised within the Bangladeshi immigrant community. The Stitches in Time artists, with a combination of professional expertise and contextual knowledge of the local community, were essential to this.

### Diversity and engaging with marginalised groups

One of many problems affecting patient engagement with care is that, necessarily, the spoken language is privileged over other forms of communication. Encouraging patients to express themselves in other ways, or using other platforms to trigger verbal expression allows an expanded range of experience to emerge. Social experience is multi-dimensional and we need to encourage imaginative ways of thinking to develop new ways of interrogating and understanding the social worlds of others [11]. Much attention is paid to the need for clinicians to listen, but often less is paid to the constraints in the patient’s ability to easily express their needs in the first place. This can be problematic, particularly when, as is common, the patient comes from a very different background to the clinician. For example, a recent study of diabetes consultations found that the patients who required interpreters were characterised by both a cultural and a social distance from the healthcare professional [12,13]. The larger social distance was reflected in limited use of humour and empathic communication, whereas patients with fluent English commonly shared jokes with the clinician, and provided stories to account for lapses in their self-management.

The most effective ‘arts in health’ projects are those which identify and articulate local issues [10]. Our project was based in Tower Hamlets, east London, a socially deprived multi-ethnic area, with over 50% of the population being from ethnic minority populations, mainly Bangladeshi [14]. Discussions in the sewing workshops revealed that many Bangladeshi women do not have a shared common language for ‘going out in the sun’ or ‘taking the children to the park’. Working on a textile for a public place about vitamin D created a framework to allow the emergence of a common discourse. This can be viewed as a form of ‘bridging work’, by which the gap between the culture of medicine and patients’ health concepts and life worlds can be negotiated [15]. Such bridges allow for innovation and flexibility to reflect local public health needs and community concerns.

### Making space for the story

We describe a project which illustrates the tension between the transactions of science and the hermeneutic narrative of people’s lives. Casting a spotlight on this can illuminate current preoccupations such as ‘holistic assessment’ and a general desire to go beyond the theme of ‘industrialised medicine’ where performance measures such as the NHS outcomes framework [16] are put centre stage. Patients attending primary care consultations usually have a story to tell [17]. However, although stories may be started, the space available for them is increasingly constricted and their value marginalised within a model of care that privileges speed and attributes which can be easily measured over continuity and narrative development. This has obvious implications for patient engagement. For example, exploring lifestyle change requires engagement with the patient’s life, and this needs to be more than a superficial inquiry. There is a corresponding need for clinicians, who are also losing the time and space for their stories to be heard in the opportunities presented by ‘coffee chats’ [18], case discussions, and clinical supervision [19]. These essential aspects of clinical praxis are increasingly fighting for air in a pressurised national health care system.

What are the new ways in which we can legitimate providing a space for the patient’s story in primary care? One model which has potential to make the narrative of people’s lives more visible is ‘social prescribing’. Social prescribing in primary care is a way of expanding the therapeutic options available to primary care consultations such that ‘new life opportunities’ may be offered to the patient ‘that can add meaning, form new relationships, or give the patient a chance to take responsibility or be creative’ ([20], p.454). This conceptualisation of social prescribing, as an extension of the exploration which starts in the consulting room, may be one way of increasing the visibility of the narrative of care linked to the patient’s life world.

Doctors are ‘agents of change, from disease to health, from brokenness to a more connected, responsive, and responsible whole’ ([21], p.2). Well-organised arts and other community projects can play a part in facilitating clinical agency, through allowing connections to be made horizontally across health, wellbeing and clinical care. Through collaborative processes they can bring into relief the individual’s health narrative and, through bridging work, enable the story to emerge in an integrated way.

Without this bridging work, a doctor lacks the cultural tool to incorporate her patient’s assumptions (e.g. about food and nutrition or about the relationships between doctors and minority patients) and habitus (e.g. predispositions for feeling ashamed about own life conditions or feeling reluctant to learn new things from a medical office) into the voice of medicine, rendering it difficult to (at least partially) expand and transform the latter from within ([15], p.499).

In our project we have seen how, when the space is provided, people share stories about health and lifestyle in group activities where health is a focus. Enabling these life world experiences to be recounted in consultations can provide opportunities for discussion about pertinent aspects of care such as behaviour change. Considering care from a horizontally oriented, rather than a vertical perspective, allows us to view human relationships in a social context and their relevance to care processes, and ultimately outcomes, becomes apparent.

### Measuring and valuing health

Community arts in health projects have a long history [22] and their value in improving people’s health and enhancing their overall well-being and confidence to self-care is acknowledged [9,10]. But in general these endeavours are seen as marginal or incidental to clinical care. In part this is related to the complexity and cost of measuring benefits, particularly if outcomes are conceived solely in biomedical terms [20].

The evaluation of community arts projects is a challenge because of the often ‘emergent, innovative and experimental nature’ of such work ([9], p.39). A recently published development and research framework for arts in health projects draws upon the UK Medical Research Council guidance on the evaluation of complex interventions [23]. This framework acknowledges the need to be sensitive to the local context, and to complexity in the way in which arts projects trigger multi-layered outcomes which can be difficult to capture. An example of a successful evaluation shows that involvement in community arts projects can be linked to wider impact on families, such as ‘healthy mothering’ [9].

The value of participation in our project can be easily described but is less easy to measure. For example, knowledge gained about healthy eating, vitamin D, and exercise may have an impact on the women’s families indirectly through discussion and behaviour changes – but is difficult to discern in the noise of a busy interconnected group of lives. The women participating in the sewing workshops, in sharing ideas and questions about health and healthcare, are mothers and carers. They indicated that they will take what they have learned to their families and friends, influencing family and community health. In other words they will participate in building social capital and social support which we know are linked to better health for individuals and communities [24,25].

## Summary

Through this interdisciplinary art project involving academics, health professionals and the local community we have become more sensitised to conceptualising one aspect of health care quality as ensuring a ‘space for the story’ in healthcare encounters. This space gives precedence to the patient narratives, but acknowledges the importance of enabling clinicians to have time to share stories about care.

This new domain of quality resonates with the concept of ‘craft knowledge’ of general practice [5] which goes beyond factual knowledge. This was originally described by Aristotle as *techne* (skill) and *phronesi*s (a form of practical wisdom) which are to be sought alongside *episteme* (factual knowledge) [26]. Horizontally oriented conceptions of quality in healthcare can incorporate connections between patients, clinicians and community, allowing narratives to emerge at different levels. These connections may be stifled if care quality is solely constructed in biomedical terms. Quality in healthcare needs to encompass the processes, benefits and value associated with narrative, made visible through good description, to enable us to build upon these rich sources of knowledge and experience.

## Endnotes

^a^For an example of the potential complexity of patient care in the current healthcare system, see ‘Holistic assessment of person with OA’ (p.11) National Institute for Health and Care Exellence: Osteoarthritis: care and management in adults. 2014. http://www.nice.org.uk/guidance/cg177/resources/guidance-osteoarthritis-pdf.

^b^See the website for the Stitches in Time project at: http://www.stitchesintime.org.uk/.

## Additional files

Additional file 1: Photograph 1. Workshop in progress: design table. Additional file 2: Photograph 2. Completed art work: vitamin D.
