# Supplementary figures and images for: Unmasking quality: exploring meanings of health by doing art
Source: BMC Fam Pract. 2015 Feb 27;16:28. doi: 10.1186/s12875-015-0233-x (PMC4343066; doi:10.1186/s12875-015-0233-x)

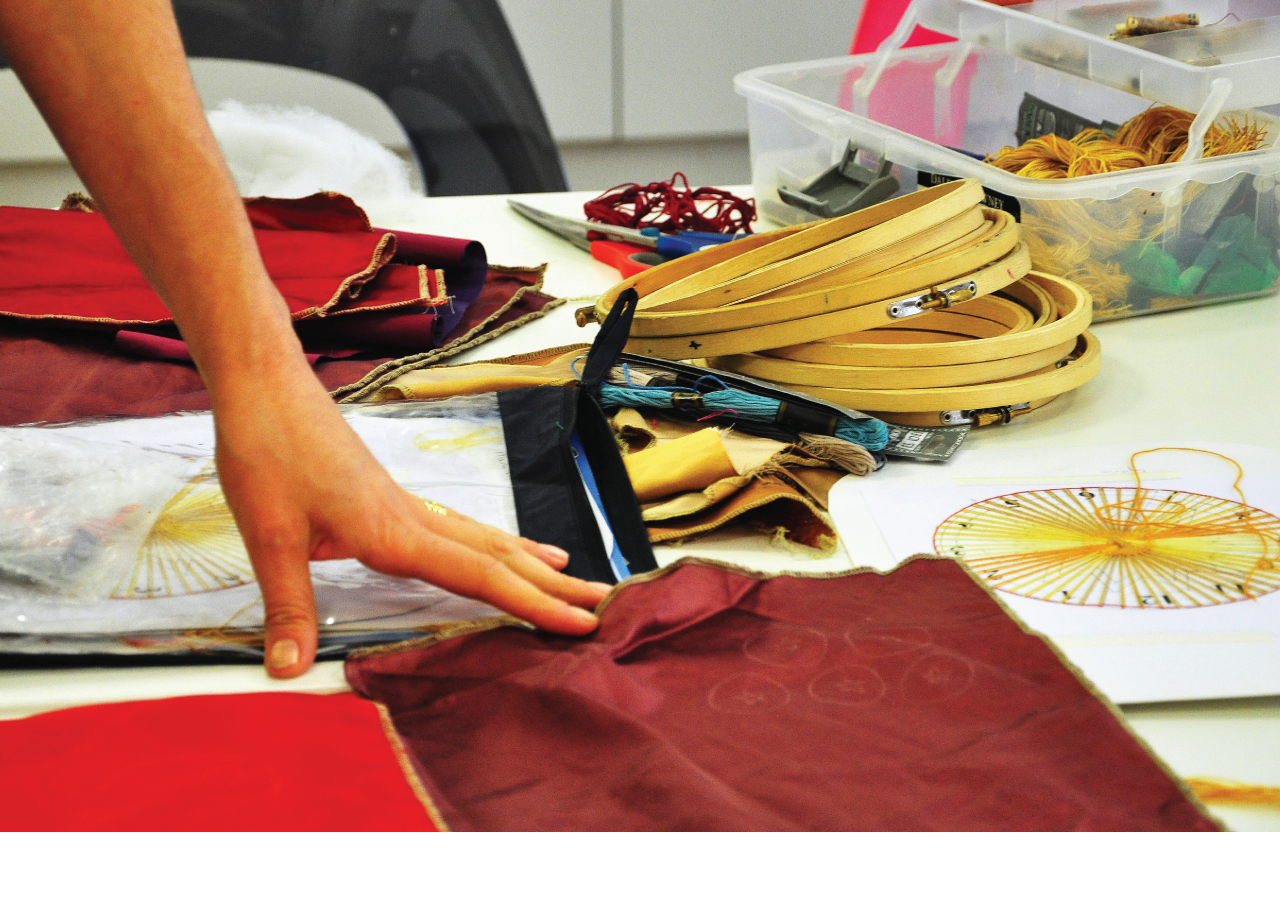

Supplement: Additional file 1: Photograph 1. — Workshop in progress: design table. [file 12875_2015_233_MOESM1_ESM.png]

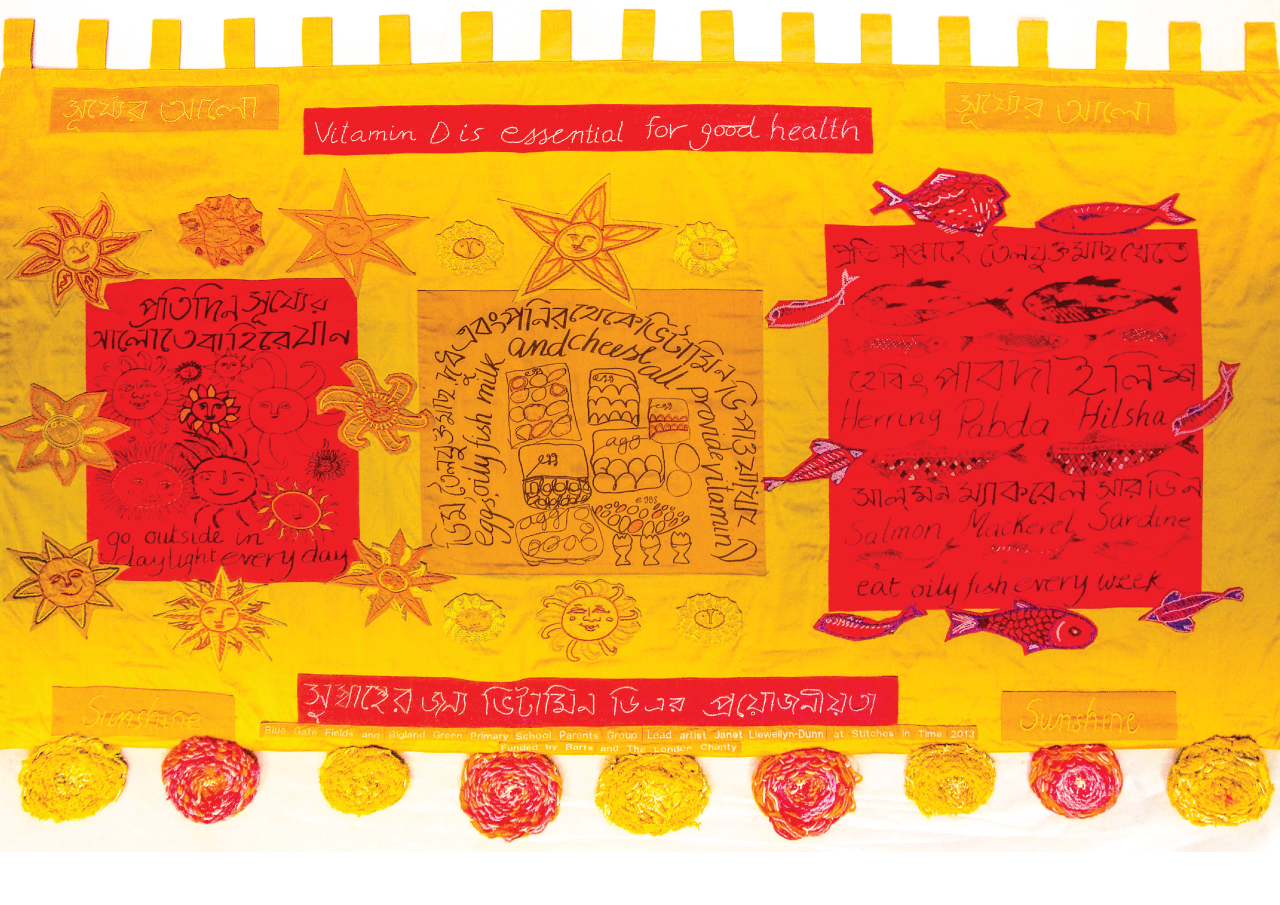

Supplement: Additional file 2: Photograph 2. — Completed art work: vitamin D. [file 12875_2015_233_MOESM2_ESM.png]
